# Class IV Lupus Nephritis in the Setting of Serologically Quiescent Disease and Normal Urine Sediment in a Patient with Late-Onset Systemic Lupus Erythematosus

**DOI:** 10.1155/2019/1219529

**Published:** 2019-02-17

**Authors:** Benedicta Nnodum, Lauren Dudley

**Affiliations:** Berkshire Medical Center, Pittsfield, MA, USA

## Abstract

Systemic lupus erythematosus (SLE) is a chronic inflammatory autoimmune disease that may affect any organ of the body. Lupus nephritis (LN) is a frequent and serious complication of SLE. We report a case of an 80-year-old woman who was initially diagnosed with late-onset SLE and eventually developed LN in the setting of normal complements, double-stranded DNA, C-reactive protein, erythrocyte sedimentation rate, and urine sediment. She developed abnormal renal function (creatinine of 1.7 mg/dl) and mild proteinuria (1-2+) without hematuria. Renal biopsy showed class IV lupus glomerulonephritis, active and chronic. The patient was started on mycophenolate mofetil which led to improvement of proteinuria and stabilization of creatinine. The suspicion for LN in a patient with late-onset SLE should remain high when there is development of suspicious renal or urinary abnormalities even if laboratory values do not suggest high disease activity and urinary sediment is normal. To our knowledge, this is one of the oldest patients with biopsy-proven LN and late-onset SLE.

## 1. Introduction

Systemic lupus erythematosus (SLE) is a chronic inflammatory autoimmune disease that may affect many organs of the body such as the skin, joints, kidneys, nervous system, heart, lungs, and the serous membranes [1]. Late-onset SLE refers to a specific group of SLE that begins above 50 years [2] and infrequently causes lupus nephritis (LN). LN significantly increases the morbidity and mortality of SLE patients and requires aggressive immunosuppressive therapy [3]. Prompt diagnosis and treatment are critical for improvement in patient survival, as evidenced by marked improvement in 5-year survival rates from 44% in the 1950s to 95% over the last 50 years [4]. Existing urine and serum biomarkers used to clinically assess patients' disease activity and predict the presence of lupus nephritis include proteinuria, urine sediment activity, creatinine clearance, anti-double-stranded DNA antibodies (anti-dsDNA Abs), complement levels C3 and C4, ESR, and CRP [5]. Nevertheless, the correlation between these markers and LN is imperfect, and their utility in reflecting disease activity and in predicting outcome remains controversial [6]. It is important to note that both C-reactive protein (CRP) and erythrocyte sedimentation rate (ESR) are often elevated in SLE flare. We present a case of a 80-year-old female with late-onset SLE and subsequent class IV lupus nephritis which developed in the setting of normal anti-dsDNA Abs, complement levels C3 and C4, ESR, CRP levels, and an inactive urine sediment.

## 2. Case Description

An 80-year-old woman with hypertension, chronic obstructive pulmonary disease (COPD), hypothyroidism, hyperlipidemia, congestive heart failure (CHF), and long-standing history of Raynaud's was diagnosed with late-onset SLE 2 years prior to the onset of lupus nephritis. At the time of diagnosis, her disease manifestations included subacute cutaneous lupus erythematosus (SCLE), pancytopenia, Raynaud's with nail-fold capillary changes, sicca symptoms, and photosensitivity. Evaluation for etiology of pancytopenia with bone marrow aspiration/biopsy with flow cytometry and cytogenetic studies, laboratory profile, and CT chest/abdomen/pelvis did not show evidence for primary bone marrow stem cell process or malignant lymphoproliferative disease. Around this time, she developed erythematous plaque lesions with scaling on her upper extremities, and biopsy findings were thought to be consistent with SCLE.

Medications included amlodipine for Raynaud's and Restasis for dry eyes. Family history was notable for a grandson with Crohn's disease but no other autoimmune diseases. Social history was remarkable for secondhand smoke exposure but no active smoking, alcohol, or drug usage.

The pertinent diagnostic tests at the time of SLE diagnosis which were positive include anti-nuclear antibody titer 1 : 1280 finely speckled, elevated rheumatoid factor (RF) of 456 IU/ml (N: 0–20 IU/ml), positive SS-A/Ro > 8.0 and SS-B/La > 8.0 antibodies, anti-beta-2 glycoprotein IgM Ab >100 U/ml, CRP level 3.9 mg/l (N: 0–5 mg/l), and ESR 51 mm/hour (N: 0–20 mm/hour). The serologic tests that were negative include anti-dsDNA Abs, complement C3 level 154 mg/dl (N: 106–194 mg/dl), complement C4 level 38 mg/dl (N: 19–50 mg/dl), cyclic citrullinated peptide IgG, cryoglobulin, serum immunofixation, Scl-70 scleroderma, Smith, RNP, cardiolipin antibodies, and lupus anticoagulant. Urine studies at the time revealed 2 + proteinuria, no hematuria or pyuria, urine protein to creatinine (PC) ratio of 0.2 (N: 0.0–0.1), and creatinine level of 0.93 mg/dl and eGFR >60 ml/min/1.73 m^2^.

**Figure 1:**
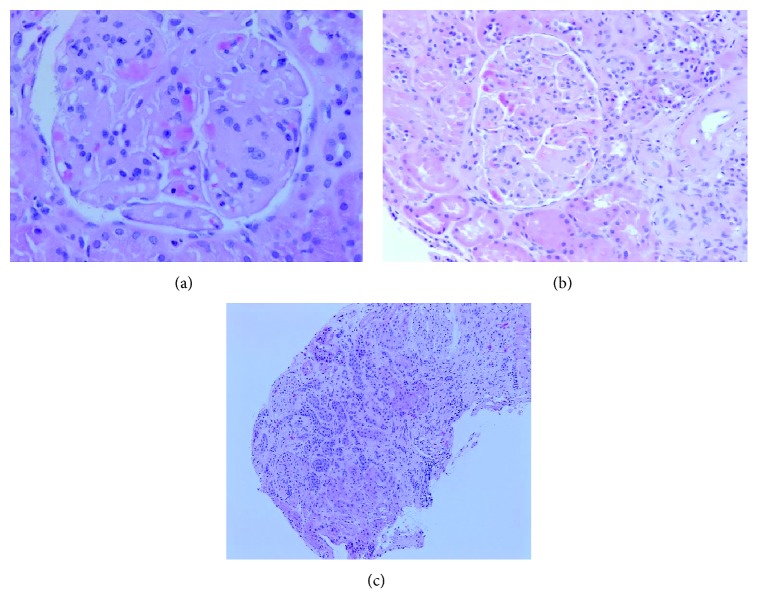
(a) Mild, focal mesangial hypercellularity, lobular architecture, and expanded matrix. The glomerular capillary wall is markedly and uniformly thickened. (b) Arteries are present and have mild arteriosclerosis. (c) Tubular atrophy involving less than 5% of the cortex.

**Figure 2:**
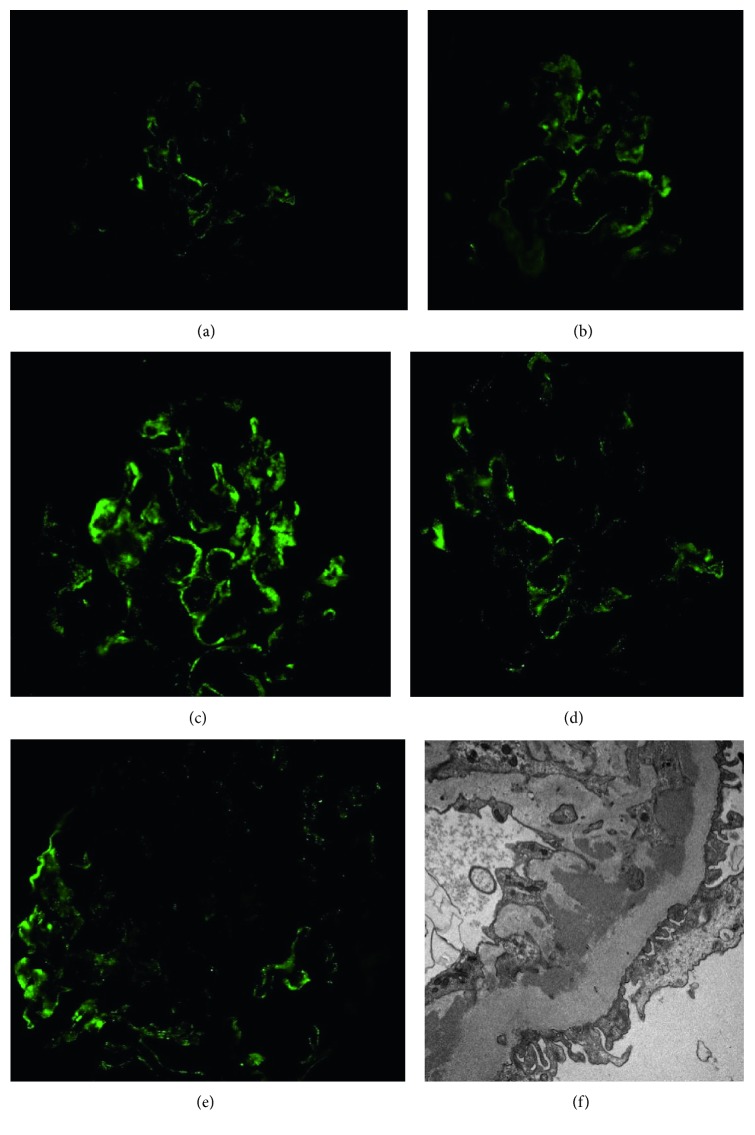
Immunofluorescence pathology slides. Mesangium showing focal moderate granular staining along the (a) GBM IgG (trace to 1+), (b) GBM IgA (1+ to 2+), (c) GBM IgM (4+), (d) GBM C1Q (2+ to 3+), and (e) GBM C3 (1+ to 2+) and (f) electron microscopy slide. No globally sclerotic glomeruli, no significant podocyte foot effacement (about 10%), numerous amorphous electron dense deposits are predominantly subendothelial.

About 2 years after the diagnosis of late-onset SLE, she was admitted and managed for a CHF exacerbation. During this hospitalization, she developed acute kidney injury (AKI) with creatinine increasing to 1.92 mg/dl during active diuresis. However, the creatinine level never came back to the baseline leaving her with a new baseline of 1.7 mg/dl and eGFR of 28 ml/min/1.73 m^2^. The etiology of the AKI was thought to be due to use of diuretics during CHF exacerbation or use of contrast during that hospitalization; however, there was a concern for the development of LN. This prompted repeating urine studies showed 4 + protein on urinalysis, worsening urine PC ratio of 1.9, and 24-hour urine protein of 1170 mg/24 hour (N: 50–150 mg/24 hour). At the time of diagnosis, there was no hematuria, an inactive urine sediment, negative dsDNA, normal ESR and CRP, and normal C3 and C4 levels. There were no symptoms or exam findings consistent with active lupus. Kidney biopsy revealed lupus nephritis, International Society of Nephrology/Renal Pathology Society class IV, active and chronic with the following findings: mild, focal mesangial hypercellularity, marked thickening of the glomerular capillary wall, segmental duplication of the glomerular baseline membrane in >50% of glomeruli, and a small focal area of interstitial nephritis with a mononuclear infiltrate. There was no necrosis, crescents, vasculitis, or focal segmental glomerulosclerosis (Figure 1). Immunofluorescence showed focal segmental granular staining along the glomerular basement membrane (GBM) for IgG (trace-1+), IgM (4+), IgA (1-2+), Kappa (3-4+), lambda (3-4+), C1q (2-3+), and C3 (1-2+). Electron microscopy showed global glomerular capillary wall thickening, GBM duplication, and subendothelial deposits with no significant podocyte foot effacement (Figure 2).

Upon diagnosis of lupus nephritis, she was started on 60 mg of oral prednisone and mycophenolate mofetil alongside atovaquone for *Pneumocystis jiroveci* pneumonia prophylaxis (sulfa allergic). Repeat urine studies after 4 weeks of being on treatment showed no proteinuria, urine PC ratio of 0.7, and improvement in creatinine to 1.4 mg/dl. She unfortunately died from infection as a complication of therapy despite renal improvement after 2 years of lupus.

## 3. Discussion

Systemic lupus erythematosus (SLE) is a chronic, inflammatory disease that can affect any organ. It is more prevalent in women than men across all ages. It is of unknown etiology and manifests by the deposition of pathologic autoantibodies and immune complexes into the tissues and cells [1]. Late-onset SLE is a well-described entity seen in older patients above 50 years accounting for 20.4% of SLE diagnoses [2]. They generally have a more benign disease course though with poorer prognosis due to higher frequency of comorbid conditions and higher organ damage in the setting of aging and higher vascular risk factors [7]. Late-onset SLE usually has lower frequency of lupus nephritis (LN) and malar rash but higher frequency of sicca syndrome, pericarditis, and lung involvement as initial manifestations [7, 8]. The atypical presentation with sicca syndrome, pericarditis, and lung involvement often makes it a challenging diagnosis to make in this age group as these symptoms can be attributed to common comorbid conditions seen in elderly patients [9, 10]. This explains the reason time from symptom onset to diagnosis can be 60 months for late-onset SLE compared to 19–24 months for adult-onset SLE [9, 10]. Recent studies that investigated the clinical characteristics between late-onset SLE and adult-onset SLE (18–50 years old) demonstrated that the former has a lower disease activity, decreased renal involvement, and lower cumulative SLE criteria [26, 27]. However, there are varying opinions on the prognosis and mortality as one study showed more favorable result [26] compared to the other [27]. Of note is that the oldest reported age of a patient diagnosed with late-onset SLE and lupus nephritis is 74 years [28]. Our patient was diagnosed with late-onset SLE at 77 years and developed stage IV LN at 79 years.

The presence of lupus nephritis is a major determinant of SLE prognosis [1]. Despite the improvement in the 5- and 10-year survival rates of LN patients, prognosis is unsatisfactory [4]. The manifestations of LN range from asymptomatic urinary findings to nephrotic syndrome and progressive renal impairment. International Society of Nephrology and the Renal Pathology Society (ISN/RPS) classes I and II usually have an indolent course while classes III, IV, and V are often progressive. Class IV LN has been shown to have a 20% 5-year probability of developing end-stage renal disease (ESRD) [11].

Some studies have shown that late-onset SLE patients have increased prevalence of positive RF, hypocomplementemia, anti-Ro/anti-La antibodies [12]. The index patient had elevated RF and anti-Ro/anti-La antibodies. The value of anti-dsDNA Ab is unclear in late-onset SLE as studies have shown mixed results [13]. Sassi et al. and Choi et al. found decreased anti-dsDNA Ab in late-onset SLE [12, 14]. However, an older study on Brazilian patients found slightly higher anti-dsDNA Ab levels in late-onset SLE [15]. Late-onset SLE has a higher incidence of elevated creatinine and decreased creatinine clearance (CrCl) on initial presentation compared to adult-onset SLE. Average CrCl of late-onset SLE was 49.1 ml/min compared to 71.2 ml/min for adult-onset SLE [16]. This is due to a combination of natural loss of renal function with age and increased incidence of hypertension at disease onset [16]. However, nephritis and nephropathy were of lower frequency [10, 12, 14]. Sassi et al. carried out a study on SLE patients that demonstrated nephritis in 26% of late-onset SLE compared to 39.8% of adult-onset SLE [14].

Renal biopsy is an invasive procedure for making a diagnosis of lupus nephritis. Noninvasive biomarkers used to predict the likelihood of lupus nephritis and select appropriate patients for biopsy include urinary protein levels, urinary sediment, complement levels C3 and C4, anti-dsDNA antibodies, and creatinine clearance [3, 5]. The rather insidious onset and fluctuating nature of LN can make early identification difficult [6]. Anti-dsDNA and complement have found to be good markers of disease activity and predictors of outcome in lupus nephritis in some studies [17]. Hsieh et al. carried out a study examining the sensitivity and specificity of these biomarkers which showed the following results: sensitivity and specific for anti-dsDNA was 80–88.6% and 92.3–97.7%, respectively, for decreased C3 level was 64.1 and 88.4%, respectively, and for decreased C4 level was 51.3 and 95.3%, respectively [17]. Anti-dsDNA was found to be the most sensitive and specific biomarker in LN. Although proteinuria measured in 24-hour urine samples or the urine PC ratio is the principal urinary biomarkers for assessing LN, they do not necessarily relate to the histological changes in LN [18]. The presence of acanthocytes or erythrocytes in the urine sediments of patients with proliferative lupus nephritis has been shown to have an 82% positive predictive value (PPV) and 71–85% negative predictive value (NPV), respectively [19]. Interestingly, low total complement hemolytic activity (CH_50_) and decreased C3 and C4 levels have been found in about 75% of SLE patients with focal nephritis and 90% in patients with diffuse nephritis [20].

Huerta et al. carried out a case series on 4 four adults with renal-limited lupus-like glomerulonephritis, two of them had positive ANA but none had low complement levels or positive anti-dsDNA Abs like our patient. They remained serologically silent with occasional occurrence of low titer of ANA despite follow-up for 8 months to 3.5 years [21]. Our patient is a case of late-onset class IV lupus nephritis occurring in the setting of low disease activity, normal inflammatory markers, complement levels, dsDNA, and urinary sediment.

Since currently available biomarkers for lupus nephritis can miss patients such as ours, renal biopsy remains the gold standard for diagnosis and should be performed in patients with SLE who have abnormal renal function or significant proteinuria. There is ongoing research for more sensitive urine biomarkers in LN as urine is easily accessible and directly shows the ongoing pathological state of a kidney through the excretion of immune-related molecules into urine [17].

The recommendation for induction treatment in patients with class IV LN is either oral/intravenous (IV) cyclophosphamide or mycophenolate mofetil (MMF), along with intravenous pulses of high-dose glucocorticoids followed by initiation of oral glucocorticoids [22]. Recent maintenance trials in LN have shown an increased number of patients attaining complete remission and fewer renal flares when MMF is used for at least 3–5 years [23]. Five-year survival rates for late-onset SLE are between 72 and 84% [10, 24], and mortality seen in this group is partly explained by comorbidities at disease onset especially hypertension, altered immune function, and decreased tolerance to immunosuppressive therapy [16, 24, 25]. In summary, majority of patients with class IV lupus nephritis have hematuria, active urine sediments, abnormal complements, positive dsDNA titers, and high measures of disease activity but our patient did not. Late-onset SLE patients are more likely to die from treatment-related complications such as sepsis [10, 24] which is what our patient died from.

## 4. Conclusion

We reported one of the latest onset cases of lupus nephritis in the literature with class IV lupus nephritis in the setting of serologically quiescent disease and normal urine sediment. Clinical suspicion for lupus nephritis must remain high when a patient with SLE develops proteinuria or abnormal renal function regardless of age or disease activity.

## Additional Points

In patients with systemic lupus erythematosus who develop abnormal renal function or proteinuria, there should be a high level of suspicion for lupus nephritis regardless of disease activity or patient's age.
